# Monograph on Diphtheria

**Published:** 1879-04

**Authors:** 


*A Monograph on the Treatment of Diphtheria based upon a new Etiology and Pathology.* By WILLIAM C. REITER, A. M., M. D. J. B. Lippincott & Co., Philadelphia.

We can best indicate the views of our author by brief extracts from the chapters upon Etiology and Treatment, though the entire paper requires to be read to gain knowledge of the author's views:

ETIOLOGY.—"Epidemic diphtheritis belongs among the infectious diseases, and even among those that are most typically *contagious*. The *miasmatic* origin of the disease is doubtful, at least in our country, where it has only occurred during the past ten years, and has appeared almost exclusively as more or less extensive epidemics which occasionally spread around from one place to another. The contagion is contained in the false membrane and shreds of tissue detached from the fauces, and in the air breathed out by the patient." . . .*

My own observations in the epidemics of 1863, '64, and '65, and in that of 1877, compel me respectfully to object to the conclusions of this most learned authority in practical medicine. The predisposing cause, whatever it be, is neither infectious nor contagious. An epidemic predisposing a people to diphtheria, erysipelas, typhoid fever, or any other disease, only attacks a proportion of the population, and our explanation is, the perfection of the vital organism resists external mal-influences. Who has not vaccinated a child for the fourth or fifth time before the poison has induced its specific effect? Whatever the predisposing epidemic cause may be, when it reaches a subject and makes its power felt, the metamorphosis of fibrin in the portal system is impaired, and the functional disturbance must be the remote cause of this fearful malady. And the violence of disease is in proportion to the fibrinous excess in the blood. How this happens is hard to tell, and impossible to explain; we live in opposition to, and in spite of, the common forces of matter.

TREATMENT.—"Prophylaxis requires that the physician should protect himself from contact with the false membrane and shreds of tissue that are coughed up, and that he should warn the attendants on the patient of the dangers of this contact. When circumstances permit, those who have nothing to do with the care of the patient should keep out of the sick-room.

"The recommendations of the varied internal and external remedies that are said to have proved efficacious against diphtheritis, have usually originated in the last stages of epidemics, at which time the cases are milder and recoveries more frequent, even without treatment. Almost all physicians experienced in the treatment of diphtheria agree that, in severe attacks, the most prized remedies *are perfectly useless*."

* Niemeyer, Practical Medicine, art, Diphtheria, vol, ii, p, 614, *et seq*,

